# From classroom to community: leveraging teacher professional development research to build capacity in community-based organizations

**DOI:** 10.3389/fpubh.2026.1819530

**Published:** 2026-05-08

**Authors:** Shinelle Kirk, Maggie Weese, Jen Cruz, Arthur Eisenkraft, Morgan Mulhern, Shoba Ramanadhan

**Affiliations:** 1 Conservation Law Foundation, Boston, MA, United States; 2 Department of Social and Behavioral Sciences, Harvard TH Chan School of Public Health, Boston, MA, United States; 3 Center of Science and Math in Context, University of Massachusetts Boston, Boston, MA, United States

**Keywords:** capacity-building, community-based organizations, evidence-based interventions, health equity, professional development

Community-based organizations (CBOs) are the trusted front line of public health, uniquely positioned to address health inequities (1). However, they face many barriers in finding and using an important tool for increasing impact: evidence-based interventions (EBIs). These barriers include limited capacity to use EBIs, conflicts between service delivery and infrastructure investment, competing priorities, lack of organizational support, and difficulty sustaining resources for EBIs (2–6). A major barrier for CBO practitioners to use EBIs is a lack of access to opportunities to build the knowledge and skills needed to successfully implement EBIs (7). While many CBO staff members have rich knowledge of communities' strengths and needs, they often lack sufficient training in public health theory or evidence-based practice (4).

Current efforts to build CBO capacity or skills, motivations, knowledge, and resources (8) for EBI use are often insufficient. Our recent work highlights three important disconnects. First, there is a lack of consensus on targets for capacity-building efforts (7). Second, there are important gaps between current capacity-building offerings and CBO practitioners' needs, which include content that emphasizes health equity, centers' practice-based, and engagement of local experts (9). Third, there is a misalignment between priority skills (one of the targets of capacity-building) between academics developing interventions and practitioners receiving them (10).

To address these disconnects, we need fresh insights. We turn to the rich education literature where similar tensions have been identified and professionals are mission-driven, work on the frontline, and often operate in resource-constrained environments. Our goal is to connect advances in capacity-building from public health (e.g., the work of Leeman et al. (8)) and insights from the field of teacher professional development (PD) (Figure 1). As an outcome of ongoing high-quality PD, practitioners' capacity for implementing EBIs is expected to increase, ultimately resulting in increased impact on the health and wellbeing of their communities. Although there have been successes in capacity-building for EBI use in CBOs, such as the Getting to Outcomes program (11) new perspectives are needed to better bridge research-and practice-based expertise (7).

**Figure 1:**
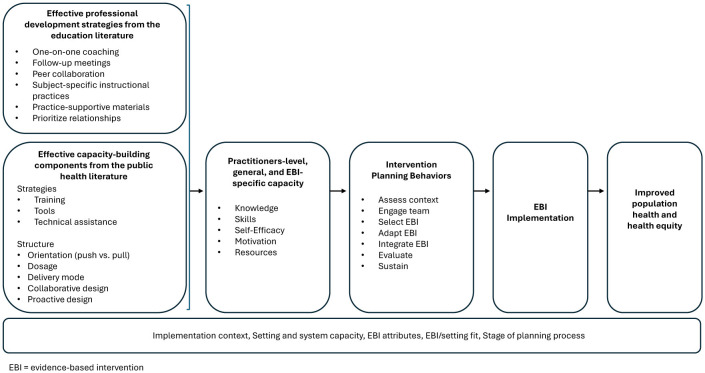
Conceptual links between practitioner capacity and health outcomes, adapted from Leeman et al. (8).

We used Hill's and Papay's synthesis of recent reviews and meta-analyses that examined the methods and content used for effective PD (which they refer to as Professional Learning), highlighting a series of actions with a strong evidence base (teacher-to-teacher collaboration, one-to-one coaching, follow-up meetings) and those with a growing evidence base (subject-specific instructional practices, concrete instructional materials, relationships with students) (13). We recognize that there are multiple models of PD in the field of education including Desimone's (13) outline of the essential features of PD, but selected this one for its grounding in the evidence base and emphasis on instructional improvement, an important analog to efforts to deliver EBIs. Below, we describe each of the core design features of PD as described in the synthesis and provide exemplar applications to capacity-building in CBOs for each factor.

1. Teacher-to-teacher collaboration

PD emphasizes the value of clear, defined teacher-to-teacher collaborations. This may involve practice-based learning opportunities, peer observation and feedback, and efforts to improve student and family engagement (12). As an example, learning communities have been highlighted as an effective strategy to build individual and group capacity (12, 14–16). Regardless of the structure, the core function served by these collaborations is that teachers are able to build relationships grounded in shared interests and goals, thereby creating a culture of empowerment that reduces isolation and emphasizes both teacher and student success. (12). This in direct contrast to short windows of opportunities where teachers may leverage their community for day-to-day support and assistance.

*Considerations for CBO capacity-building*: Parallels to teacher-to-teacher collaborations can be found in CBO capacity-building efforts in the forms of community collaboratives and communities of practice. For CBO practitioners, this unique approach to capacity-building can provide continuous opportunities to engage in problem solving, with additional benefits of relationship building, information sharing, and an increase in self-efficacy (17), though the evidence is mixed on the impact of these collaborations (18). A more foundational consideration comes from grounding PD in collaboration between implementers (here, CBO staff, centering their expertise, and creating lasting structures for sharing challenges, success, and co-developed solutions. This connects with the broader participatory implementation science literature in emphasizing assets held by a range of actors and supporting structures that improve systems over the long-term to advance impact and equity (19).

2. One-to-one coaching

As a tool, coaching can foster a PD environment that prioritizes teacher and student growth (20). Coaching is effective when it is an ongoing, individualized process that supports teachers to build on and demonstrate theoretical knowledge (21). Additionally, coaches can support teachers in acquiring this knowledge by providing detailed and targeted advanced lesson planning (12). While research recommends that coaching be continuous, this is a non-trivial investment. Further, one of the challenges faced in developing a coaching program is the disconnect that can occur between coaches and teachers with experienced teachers being more resistant than others (22).

*Considerations for CBO capacity-building:* Individualized coaching can be used in CBO settings to offer support once program implementation is underway (3). In this way, coaches might work closely with staff, providing guidance feedback, and expertise in EBI delivery. Key outcomes are staff empowerment and the enhancement of organizational capacity. Notably, effective coaching is contingent upon mutual trust and transparency (23).

3. Follow-up meetings

Immediate, iterative, practice-based follow-up sessions after trainings with opportunities for inquiry and feedback have been identified as an important PD strategy (12). This keeps training material readily accessible, fosters collaboration among instructors, and can increase a sense of accountability for creating practice change (12, 24). In addition, follow-up also allows trainers to evaluate and modify the program based on participant feedback.

*Considerations for CBO capacity-building:* Offering support after initial trainings, is a common strategy in public health and has been shown to effectively support PD goals (8, 11). Systematically, this ensures that CBOs are trained and supported in implementing interventions to maximize impact and sustainability. Challenges to the use of this strategy may be the ability to engage CBO practitioners repeatedly and over time as these individuals are often over-burdened and unable to attend sessions at the expense of delivering services (25). Virtual and asynchronous interactions may offer an opportunity to overcome these barriers.

4. Subject-specific instructional practice (or knowing how to teach a concept)

Subject-specific instructional practice emphasizes the importance of *pedagogical content knowledge*, or understanding what makes it easy or hard to learn certain topics, as well as the conceptions and preconceptions that learners bring into the learning environment (26). This type of knowledge refers to unique teaching approaches in a specific discipline, such as effective ways to teach motion in a physics class (vs. content knowledge, which focuses more on the laws of motions in physics) (1–5). The general assumption is that new and experienced teachers are well versed in pedagogical content knowledge, which is not always true (15). Two approaches to supporting pedagogical content knowledge come from curriculum development (e.g., helping teachers create flexible lesson plans) and peer observations (27, 28). While valuable, these strategies place a fair amount of burden on teachers and administrators to allow time and support to create clear guidelines and expectations (29, 30).

*Considerations for CBO capacity-building*: Given that many CBO staff have not had formal training in public health or delivery of EBIs, capacity-building efforts have a large impact by centering CBO practice, e.g., focusing on what it takes to adapt and implement a specific intervention. Integrating participatory approaches to implementation science and the content of the EBI identifies important opportunities for capacity-building efforts to draw from and spread practitioners' expertise in these areas (19).

5. Practice-supportive materials

The notion of practice-supportive materials is to emphasize the needs of teachers in achieving concrete teaching goals (12). In contrast to an emphasis on focusing on fundamental concepts and ideas, these materials provide teachers with relevant and accessible materials for instruction (31). Such materials may include additional lesson plans, or training in a specific model. Further, it also reduces the pressure on teachers to modify curricula, instead, providing a stronger focus on classroom instruction and student outcomes (12).

*Considerations for CBO capacity-building*: For CBO practitioners, this emphasizes the need to offer materials that prioritize practice activities, e.g., tools to support EBI adaptation, over materials that emphasize underlying content. As one example, our team offered capacity-building workshops for CBO practitioners that included case examples for which participants were provided with web-based resources, worksheets, and tools to go through the process of finding, selecting, adapting, and planning to implement an EBI (32). Similarly, the Cancer Prevention and Control Research Network offers capacity-building for EBI use that offers a rich range of practice-focused tools and materials to support CBOs in achieving practical goals (33).

6. Relationships with students

Researchers have suggested that one often overlooked aspect of professional development are teacher-student relationships (34, 35). In their analysis, Hill and Papay note that student academic outcomes are dependent on the interactions between teachers and students. Strengthening relationships among teachers and students can also reduce disciplinary actions and classroom interruptions (12).

*Considerations for CBO capacity-building:* The parallel for CBOs is connections to communities and for many CBO practitioners, a sense of mission drives their work. The literature is already clear that investing in these deep connections yields benefits via increased receptiveness to EBIs and access to available, local supports among the communities served (7). By centering the ways in which practitioners develop deep relationships with community members, PD efforts can demonstrate alignment with practitioner goals. Drawing on CBOs' strong community relationships can improve communication channels and increase uptake of evidence-based solutions (36).

## Conclusion

By experimenting with these strategies from the field of education, public health actors can move beyond merely “disseminating” information to CBOs to building a sustainable infrastructure of professional learning that respects practitioner expertise, improves EBI implementation, and ultimately advances health equity in underserved communities. In this way, capacity-building efforts in CBOs will stay true to the goal of addressing not equipping organizations to address not only current community needs, but those that will arise in the future (37). We recognize that future work must move beyond identifying core features of conceptual models and further contribute to the evidence base of strategies for PD (30). The features highlighted in this commentary offer a range of design considerations for CBO capacity-building and suggest the potential power of strategic attention to each of the features in a given capacity-building effort.
